# Emergence of satellite DNAs suggests centromeric repositioning as a driver of karyotypic variation of the freshwater darter characines (*Apareiodon affinis*)

**DOI:** 10.1007/s10577-026-09793-7

**Published:** 2026-02-20

**Authors:** Marina Soares Ribas, Matheus Azambuja, Viviane Nogaroto, Marcelo Ricardo Vicari

**Affiliations:** 1 Programa de Pós-Graduação em Genética, Universidade Federal do Paraná, Centro Politécnico, Avenida Coronel Francisco H. dos Santos, 100, Curitiba, Paraná 81531-990 Brazil; 2 Departamento de Biologia Estrutural, Molecular e Genética, Universidade Estadual de Ponta Grossa, Av. Carlos Cavalcanti, 4748, Ponta Grossa, Paraná 84030-900 Brazil

**Keywords:** Centromere, Centromeric repositioning, Chromosomal rearrangements, Karyotype evolution, Pericentric inversion

## Abstract

Repetitive DNA sequences actively contribute to karyotype diversification by accumulating mutations, exhibiting susceptibility to DNA double-strand breaks that promote chromosomal rearrangements, and reshuffling within centromeric heterochromatin, among other processes. Parodontidae shows a conserved diploid number of 54, with predominantly metacentric and submetacentric chromosomes. *Apareiodon affinis*, from the Lower Paraná River, was described with three karyomorphs due to interpopulation structural variation, characterized by four to sixteen acrocentric chromosomes. However, the mechanisms driving chromosomal variation and the contribution of satellite DNA to these processes remain poorly understood. In this study, we characterized the *A. affinis* satellitome to assess the role of satellite sequences in the diversification of acrocentric chromosomes. A total of 48 satellite DNAs were identified, and 16 of them were mapped *in situ*. The data demonstrated that intragenomic homogenization mechanisms led to alterations in satellite sequence dominance, and intergenomic mechanisms contributed to the diversification of orthologous sequences when compared with other Characiformes families. *In situ* localization revealed that most satellites in the centromeric regions of acrocentric chromosomes were absent from the centromeres of meta/submetacentric chromosomes. The satellite *Aaf*Sat01-200, which is highly diversified and the most abundant in the *A. affinis* genome, exhibits exclusive centromere localization on acrocentric chromosomes. Our results indicate the presence of distinct monomers with centromeric function in *A. affinis* and identify *Aaf*Sat01-200 as the principal element involved in the centromeric repositioning mechanism that led the expansion of acrocentric chromosomes in this lineage.

**Supplementary Information:**

The online version contains supplementary material available at 10.1007/s10577-026-09793-7.

## Introduction

The genomic content of eukaryotic species is enriched by repetitive elements (Biscotti et al. 2015). According to their origin and function, repetitive sequences are classified into gene families, transposable elements (TEs), and satellite DNAs (satDNA) (Nei and Rooney 2005; Wicker et al. 2007; Garrido-Ramos 2017). TEs and satDNA are currently considered key elements for understanding structural and functional characteristics in eukaryotic genomes (Garrido-Ramos 2017; Wells and Feschotte 2020). Structurally, repetitive DNAs organize essential regions of chromosomes, such as telomeres and centromeres (Slijepcevic 2016; Vicari et al. 2022; Enriquez and Nechemia-Arbely 2025). Additionally, repetitive sequences are considered hotspots for mutations and genomic variations, triggering polymorphisms, chromosomal rearrangements, and the origin and differentiation of sex and supernumerary chromosomes (O’Neill et al. 2004; Farré et al. 2011; Utsunomia et al. 2019; Wolf et al. 2024; de Oliveira et al. 2025).

From a functional perspective, repetitive sequences participate in various mechanisms of gene regulation, including transcriptional and post-transcriptional processes, as well as chromatin modification (Cech and Steitz 2014; Peschansky and Wahlestedt 2014; Zhang et al. 2019; Wells and Feschotte 2020). Given the importance of the repetitive fraction for genomic organization and function, satDNA characterization has gained prominence, as these sequences may act as regulatory sites, serve as hotspots for DNA double-strand breaks (DSBs), and exhibit transcriptional activity that leads to the generation of non-coding RNAs (Kuhn 2015; Garrido-Ramos 2017; Lower et al. 2018; Goes et al. 2023).

Advances in genomic analyses have led to a reevaluation of satDNA concept (Garrido-Ramos 2015, 2017; Šatović-Vukšić and Plohl 2023). The complete set of these sequences within a genome is referred to satellitome (Ruiz-Ruano et al. 2016). SatDNA is traditionally defined as long arrays of highly similar monomers arranged *in tandem* repeats (Richard et al. 2008; Plohl et al. 2012; Garrido-Ramos 2015, 2017). In general, satDNAs are located in the pericentromeric and subtelomeric regions of chromosomes and constitute a major component of heterochromatin (Plohl et al. 2012; Hartley and O'Neill 2019). Satellitomes analysis in different organisms have revealed that they can represent a highly diverse group, varying in numerous features, such as monomer length and complexity, copy number, epigenetic modifications, chromatin state, and chromosomal distribution, among others (Šatović-Vukšić and Plohl 2023).

According to satDNA library hypothesis proposed by Fry and Salser (1977), organisms from related species or genera share a common set of satellites due to their shared ancestry. Thus, differences in the size and abundance of satDNA sequences are expected to reflect evolutionary processes occurring both within lineages and among species (Fry and Salser 1977). Supporting this hypothesis, closely related species share a common satellite DNA library, although they exhibit different patterns of sequence accumulation and chromosomal distribution across lineages (Meštrović et al. 1998, 2006; Cesari et al. 2003; Koukalova et al. 2010; del Bosque et al. 2014; de Silva et al. 2017; Samoluk et al. 2017; Goes et al. 2022).

The advent of next-generation sequencing (NGS) techniques, together with the development of bioinformatics tools, has enabled the analysis of the satellitome, gene families, microsatellites, and TEs in “non-model” species (Novák et al. 2010, 2013, 2017; Ruiz-Ruano et al. 2016; Azambuja et al. 2022; Glugoski et al. 2022; de Oliveira et al. 2024, 2025). Parodontidae (Characiformes) groups three genera: *Parodon*, *Saccodon*, and *Apareiodon*, with 32 valid species (Fricke et al. 2025). Its representatives are characterized by a shared diploid chromosome number (2n) of 54 (Bellafronte et al. 2011). However, despite sharing the same 2n, parodontids vary in chromosome morphology, abundance, and distribution of repetitive elements, as well as in the differentiation of sex chromosomes, with female heterogamety reported in some species (Schemberger et al. 2011; Traldi et al. 2020; Nirchio et al. 2021; Azambuja et al. 2023).

The darter characines *A. affinis* has the La Plata River (Buenos Aires, Argentina) as its type locality but is distributed throughout the entire hydrographic system of the Lower and Upper Paraná River (Pavanelli 2003). It is one of the most intriguing species of Parodontidae in terms of karyotypic plasticity, exhibiting four karyomorphs and distinct molecular operational taxonomic units (do Nascimento et al. 2018). Specimens from the Upper Paraná River (karyomorph A) have a multiple sex chromosome system (♂ 2n = 54, ZZ - 50 m/sm + 4 st; ♀ 2n = 55, ZW_1_W_2_ - 49 m/sm + 6 st), while representatives from the Lower Paraná River exhibit demes with a variable karyotype formula and no sex chromosome heteromorphism (do Nascimento et al. 2018). In representatives from the Lower Paraná River, variation in the number of acrocentric chromosomes and structural polymorphisms in the rDNA clusters have been observed, as follows: Uruguay River 46 m/sm + 4 st + 4 a (karyomorph B); Cuiabá River 42 m/sm + 2 st + 10 a (karyomorph C); and Paraguay River 36 m/sm + 2 st + 16 a (karyomorph D) (do Nascimento et al. 2018). Regarding the distribution of satDNA, a single sequence isolated from *Parodon hilarii* and named pPh2004 (Vicente et al. 2003) was mapped to the chromosomes of *A. affinis*, showing preferential centromeric location in acrocentric chromosomes of karyomorphs C and D (do Nascimento et al. 2018).

In this context, we characterized the satellitome of *A. affinis* karyomorph D (Paraguay River) and performed comparative *in situ* localization across the three karyomorphs distributed in the Lower Paraná River to evaluate the role of satDNA in the structural organization and chromosomal evolution of the species.

## Material and methods

### Satellitome characterization of *A. affinis*

Total DNA from a male of *A. affinis* sampled in Paraguay River (karyomorph D) was extracted from the liver tissue using the Reliaprep gDNA Tissue Miniprep System kit (Promega, Madison, WI, USA). The DNA quality was checked on a 1% agarose gel and quantified using the BioPhotometer D30 (Eppendorf, Hamburg, Germany). The DNA was sequenced on the Illumina MiSeq platform using a paired-end strategy (2 × 100 bp, 10 million raw reads).

Data from the DNA sequencing of *A. affinis* were used to prospect satDNA. The preprocessing steps for the raw reads followed the default parameters recommended by Novák et al. (2020) for repetitive DNA analysis of unassembled sequence reads. The reads were pre-processed using the FASTQ paired-end reads tool (QC > 10, 100 bp) for quality control and adapter removal, avoiding selective removal of specific classes of repetitive DNA, and generating an interlaced reads library. Two random subsets of 1,000,000 reads each were then obtained using the Read sampling tool. Next, the Tandem Repeat Analyzer (TAREAN) software (Novák et al. 2017), available on the Galaxy server (Novák et al. 2013), was used to prospect the satellite sequences. The satDNA nomenclature followed the system proposed by Ruiz-Ruano et al. (2016), which consists of the species acronym followed by “Sat” (*Aaf*Sat), a number representing the sequence’s relative genomic abundance, and the length of the consensus sequence.

The obtained satDNA sequences were submitted to comparative analysis in the CENSOR (Kohany et al. 2006), Basic Local Alignment Search Tool for nucleotides (BLASTn) (Altschul et al. 1990), and Dfam (Storer et al. 2021) databases, to identify satellites previously characterized in other fish species, and to remove other sequences frequently misidentified as satellites, such as gene families.

The homology among the satDNAs was determined with RepeatMasker 4.1.3 software (Smit et al. 2013−2015), using the Search engine Crossmatch and the “rm_homology_v2.py” script (https://github.com/fjruizruano/ngs-protocols/blob/master/rm_homology_v2.py, to group the sequences into variants, families, and superfamilies, as proposed by Ruiz-Ruano et al. (2016). The “rm_homology_v2.py” script was also used to identify the presence of the pPh2004 satellite among the satellites identified in *A. affinis*. The abundance and divergence of each satellite were determined using RepeatMasker by masking the *A. affinis* genomic library (2 × 5,000,000 reads) against the catalog of satellites obtained. Repeat landscape graphs were generated, based on the Kimura-2-parameter (K2P) nucleotide substitution model, using the calcDivergence-fromAlign function.pl script with RepeatMasker (Smit et al. 2013−2015) for the *A. affinis* genome, and individually for the *Aaf*Sat01-200, *Aaf*Sat02-2918, *Aaf*Sat03-235, and *Aaf*Sat11-227 satellites.

### Probe synthesis for *in situ* localization

The 19 most abundant satellite sequences recovered from the *A. affinis* genome were selected for *in situ* localization assays. For 17 of the identified sequences, primers were designed using the Primer3Plus software (Untergasser et al. 2007) (Supplementary Material, Table S1) for sequence isolation by Polymerase Chain Reaction (PCR). *Aaf*Sat09-33 and *Aaf*Sat12-52 probes were produced by adding a 3'-end biotin label during oligo synthesis. The PCRs were composed of 0,2 - 40 ng of genomic DNA template (Table S1), 0,4 μM of forward and reverse primers, 0,2 mM dNTPs, 1 mM MgCl_2_, 1X *Taq* reaction buffer (Tris 200 mM, pH 8,4, KCl 500 mM), and 1 U of *Taq* DNA Polymerase (Invitrogen). PCRs were performed with the following parameters: 95 ºC for 10 min, followed by 35 cycles (95 ºC for 1 min, 55.1–60.2 ºC for 40 s - details in Table S1, and 72 ºC for 30 s), and a final extension at 72 ºC for 10 min (Table S1). PCR products were verified on a 1% agarose gel by comparing the amplicon size and the ‘ladder’ pattern characteristic of tandem repeats. Finally, the probes were synthesized in Nick Translation reactions using the Biotin16 NT labeling mix (Jena Bioscience, Jena, Germany) and Digoxigenin NT Labeling kits (Jena Bioscience).

### Biological samples and chromosome preparations

Cytogenetic preparations of *A. affinis* from different tributaries of the Lower Paraná River hydrographic system (Uruguay, Cuiabá, and Paraguay rivers) were used (Supplementary Material, Table S2). Mitotic preparations were obtained from the anterior kidney according to the protocol described by Bertollo et al. (2015).

### Fluorescence *in situ* Hybridization (FISH)

FISH was performed according to the protocol described by Pinkel et al. (1986), with the following hybridization conditions: 200 ng of probe, 50% formamide, 2XSSC (Saline-Sodium Citrate), 10% dextran sulfate, at 37 °C for 16 h. Signals were detected using Alexa Fluor 488 Streptavidin (Molecular Probes, Eugene, USA) and anti-digoxigenin rhodamine Fab fragments (Roche Applied Science, Penzberg, Germany). Metaphases were counterstained with 0,2 µg/mL of 4,6-diamidino-2-phenylindole (DAPI) in VECTASHIELD mounting medium (Vector Laboratories, Burlingame, USA) and analyzed under a fluorescence microscope (Leica DM 2000) coupled with a DFC3000 G CCD camera (Leica). *Apareiodon affinis* chromosomes were identified according to the arm ratio rule proposed by Levan et al. (1964), classified as metacentric (m), submetacentric (sm), subtelocentric (st), and acrocentric (a), and arranged into karyomorphs B, C, and D, according to do Nascimento et al. (2018).

### Non-B motifs annotation in the satDNAs

A-phased repeats (APR), direct repeats (DR), inverted repeats (IR), mirror repeats (MR), short tandem repeats (STR), and Z-DNA motifs were annotated with Non-B DNA Motif Search Tool (Cer et al. 2013).

## Results

**Fig. 1:**
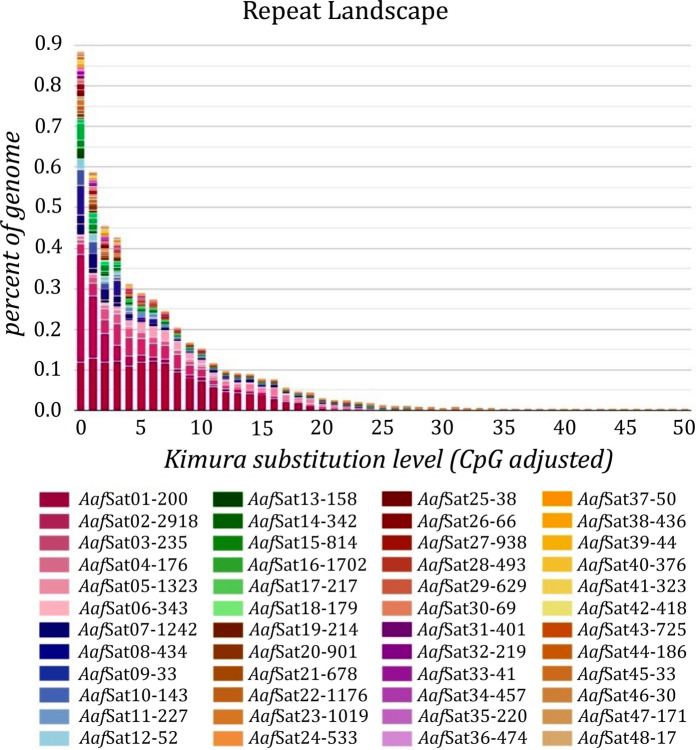
Repeat landscape graphs showing the abundance (Y-axis) and K2P divergence (X-axis) profiles of the *A. affinis* karyomorph D satellitoma

**Fig. 2:**
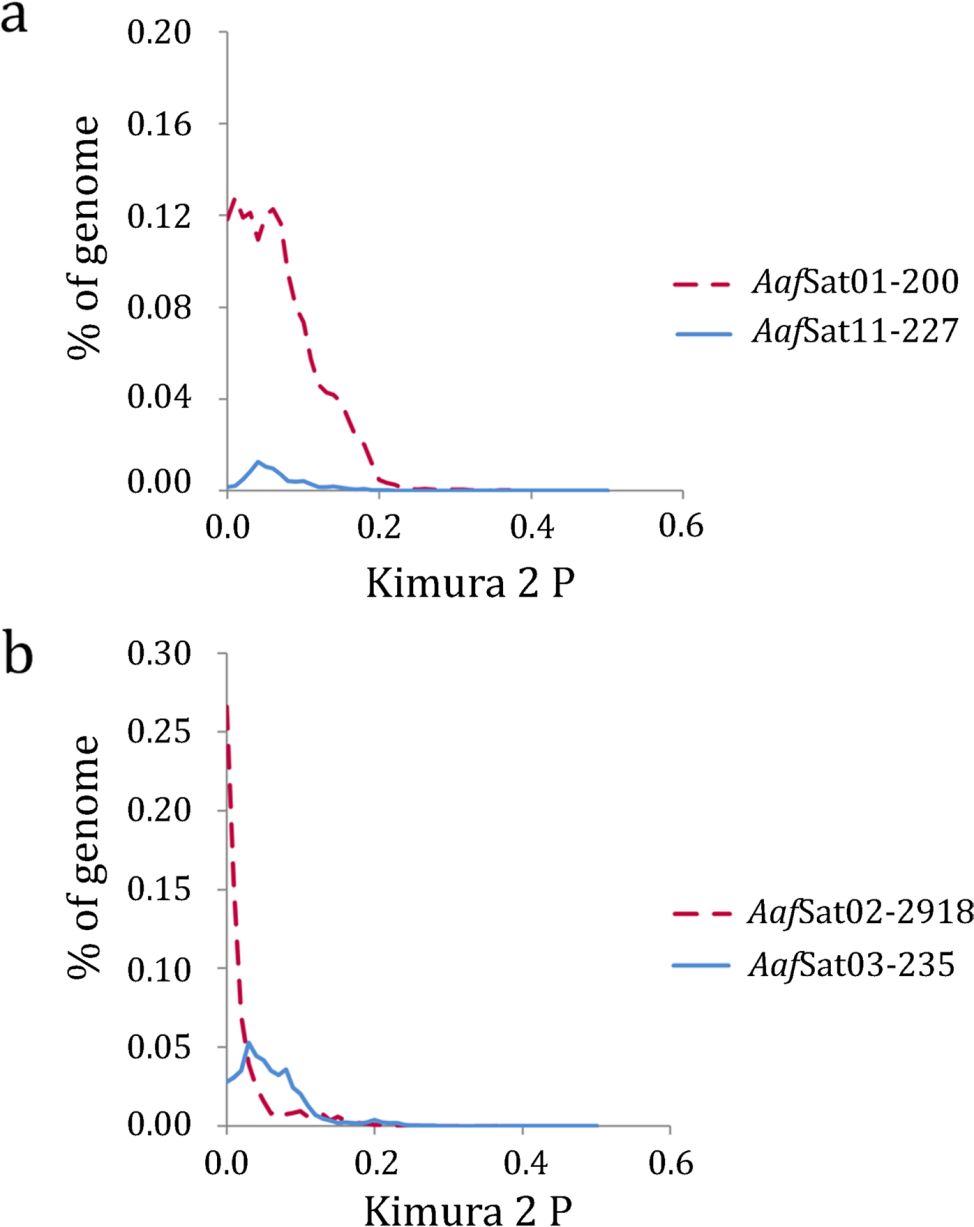
Comparative repeat landscape graphs showing the abundance (Y-axis) and K2P divergence (X-axis) profiles. In (**a**) *Aaf*Sat01-200 (red) versus *Aaf*Sat11-227 (blue); In (**b**) *Aaf*Sat02-2918 (red) versus *Aaf*Sat03-235 (blue)

After two iterations of the TAREAN tool, 48 high-confidence and 30 low-confidence satDNA sequences for *A. affinis* from the Paraguay River (karyomorph D) were identified (GenBank accession: PX685767-PX685814). Sequences classified as low-confidence were removed from the analysis because they were unlikely to represent satDNAs. The Repeat Unit Lengths (RUL) ranged from 17 to 2,918 bp, with an average size of 460 bp (Table 1). The length distribution of consensus sequences showed that long satDNA (>100 bp) is prevalent in the genome, particularly in 37 of the 48 families. The A+T content of the satDNA ranged from 44.7 to 70%, with an average value of 58.12%, indicating the predominance of A/T-rich families (Table 1). Homology analysis of the sequences grouped 16 monomers into four superfamilies (SF) (Table 1). SF1 was represented by seven satellites: *Aaf*Sat05-1323, *Aaf*Sat16-1702, *Aaf*Sat23-1019, *Aaf*Sat27-938, *Aaf*Sat34-457, *Aaf*Sat42-418, and *Aaf*Sat44-186, while SF2 grouped two sequences, *Aaf*Sat03-235 and *Aaf*Sat11-227. The satDNAs *Aaf*Sat08-434, *Aaf*Sat10-143, *Aaf*Sat13-158, and *Aaf*Sat15-814 were grouped into SF3, and SF4 was represented by the satellites *Aaf*Sat21-678, *Aaf*Sat31-401, and *Aaf*Sat38-436 (Table 1). The homology search between the pPh2004 satellite and the satDNA of *A. affinis* revealed a 73.74% sequence identity and 98% coverage with *Aaf*Sat01-200 (Table 2). A sequence similarity search identified 13 satellites in *A. affinis*, with small segments (14 to 45% coverage) exhibiting high identity (>80%) with satellite sequences from other Characiformes species (Table 2). The satellitome of *A. affinis* represents 4.84% of its genome. The Repeat landscape graph for the satDNA showed a higher abundance of satellites with lower divergence values (Fig. 1). Comparative landscape analysis revealed that the satellites *Aaf*Sat01-200 and *Aaf*Sat11-227 (Fig. 2a), as well as satellites *Aaf*Sat02-2918 and *Aaf*Sat03-235 (Fig. 2b), have diverged recently. Both *Aaf*Sat01-200 and *Aaf*Sat02-2918 exhibited a consistent expansion phase and became dominant elements in the genome. Concomitant with the expansion of these satellites, *Aaf*Sat11-227 and *Aaf*Sat03-235 stopped accumulating additional copies in the genome (Fig. 2a, b).

**Table 1:** Characteristics of the 48 satDNA obtained from the *A. affinis* (karyomorph D) genome

Satellite	Ab	SF	RUL	A + T (%)	Div (%)	Satellite	Ab	SF	RUL	A + T (%)	Div (%)
*Aaf*Sat01-200	1.7		200	58.5	7.06	*Aaf*Sat25-38	0.034		38	57.9	2.17
*Aaf*Sat02-2918	0.6		2.918	58.7	2.40	*Aaf*Sat26-66	0.032		66	63.6	4.49
*Aaf*Sat03-235	0.48	2	235	64.7	6.24	*Aaf*Sat27-938	0.031	1	938	52	0.92
*Aaf*Sat04-176	0.26		176	65.9	5.74	*Aaf*Sat28-493	0.028		493	63.7	10.73
*Aaf*Sat05-1323	0.2	1	1323	49.8	12.67	*Aaf*Sat29-629	0.027		629	56.4	7.27
*Aaf*Sat06-343	0.19		343	60.9	7.91	*Aaf*Sat30-69	0.026		69	59.4	4.15
*Aaf*Sat07-1242	0.16		1242	44.7	9.39	*Aaf*Sat31-401	0.025	4	401	50.9	2.15
*Aaf*Sat08-434	0.13	3	434	59.2	3.81	*Aaf*Sat32-219	0.024		219	58.4	8.94
*Aaf*Sat09-33	0.11		33	63.6	1.75	*Aaf*Sat33-41	0.023		41	65.9	7.30
*Aaf*Sat10-143	0.11	3	143	55.2	1.76	*Aaf*Sat34-457	0.021	1	457	51.2	1.15
*Aaf*Sat11-227	0.1	2	227	63.4	6.88	*Aaf*Sat35-220	0.018		220	55.5	6.14
*Aaf*Sat12-52	0.1		52	63.5	2.42	*Aaf*Sat36-474	0.018		474	60.3	2.98
*Aaf*Sat13-158	0.077	3	158	51.9	8.51	*Aaf*Sat37-50	0.018		50	70	2.32
*Aaf*Sat14-342	0.076		342	61.7	7.91	*Aaf*Sat38-436	0.018	4	436	61.5	2.46
*Aaf*Sat15-814	0.071	3	814	55.5	2.29	*Aaf*Sat39-44	0.018		44	56.8	2.88
*Aaf*Sat16-1702	0.068	1	1702	51.3	0.55	*Aaf*Sat40-376	0.017		376	54.3	5.08
*Aaf*Sat17-217	0.058		217	61.8	3.14	*Aaf*Sat41-323	0.016		323	68.1	4.85
*Aaf*Sat18-179	0.051		179	57	14.97	*Aaf*Sat42-418	0.016	1	418	56.5	2.10
*Aaf*Sat19-214	0.046		214	58.9	4.30	*Aaf*Sat43-725	0.015		725	64.4	1.15
AafSat20-901	0.038		901	64.6	1.35	*Aaf*Sat44-186	0.013	1	186	57	3.26
*Aaf*Sat21-678	0.037	4	678	52.4	6.08	*Aaf*Sat45-33	0.013		33	66.7	2.06
*Aaf*Sat22-1176	0.036		1176	59	1.99	*Aaf*Sat46-30	0.012		30	46.7	3.82
*Aaf*Sat23-1019	0.035	1	1019	54.3	5.88	*Aaf*Sat47-171	0.011		171	49.7	0.95
*Aaf*Sat24-533	0.034		533	59.3	3.99	*Aaf*Sat48-17	0.011		17	47.1	1.72

*Ab* relative abundance; *SF* superfamily; *RUL* repeat unit length; *A + T%* AT content; *Div* divergence

**Table 2:** Genetic similarity results among satellites of *A. affinis* and other Characiformes

*A. affinis* satellite	Aligned family	Species	Coverage	Identity
*Aaf*Sat01-200*	pPh2004	*Parodon hilarii*	98.00%	73.74%
*Aaf*Sat05-1323	*Cmo*Sat049-938	*Cyphocharax modestus*	18.00%	95.60%
*Aaf*Sat16-1702	*Pse*Sat020-1284	*Pyrrhulina semifasciata*	17.00%	100%
*Aaf*Sat20-901	*Pse*Sat033-880	*Pyrrhulina semifasciata*	17.00%	83.16%
*Aaf*Sat21-678	*Mma*Sat060-1683	*Megaleporinus macrocephalus*	26.00%	98.91%
*Aaf*Sat22-1176	*Hma*Sat034-632	*Hoplias malabaricus*	19.00%	91.49%
*Aaf*Sat23-1019	*Pse*Sat62-469	*Pyrrhulina semifasciata*	14.00%	100%
*Aaf*Sat27-938	*Mma*Sat094-450	*Megaleporinus macrocephalus*	16.00%	98.73%
*Aaf*Sat31-401	*Mma*Sat082-626	*Megaleporinus macrocephalus*	45.00%	100%
*Aaf*Sat34-457	SatCE06	*Cobitis elongatoides*	16.00%	100%
*Aaf*Sat40-376	*Mma*Sat102-330	*Megaleporinus macrocephalus*	41.00%	98.09%
*Aaf*Sat42-418	*Cmo*Sat089-1371	*Cyphocharax modestus*	18.00%	98.68%
*Aaf*Sat44-186	*Cmo*Sat059-853	*Cyphocharax modestus*	39.00%	100%
*Aaf*Sat47-171	*Pma*Sat47-568	*Pyrrhulina marylinae*	44.00%	98.68%

^*^Manually generated in BLASTn using *Aaf*Sat01-200 as the query and the pPh2004 sequence from Vicente et al. (2003) as the subject

**Fig. 3:**
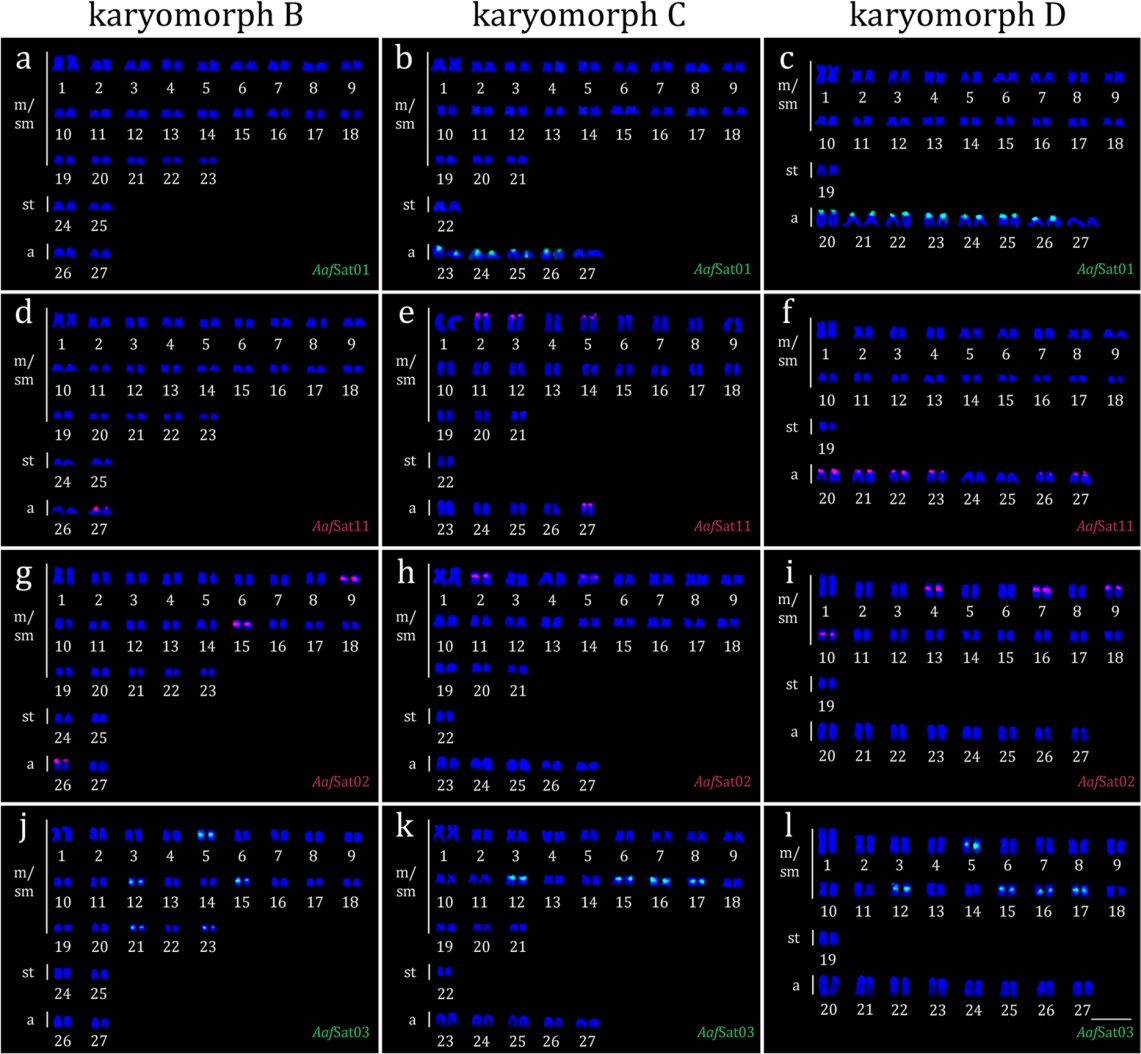
Karyotypes of *A. affinis* from the Uruguay River – karyomorph B (a, d, g, j), Cuiabá River – karyomorph C (b, e, h, k), and Paraguay River – karyomorph D (c, f, i, l) subjected to FISH with the probes *Aaf*Sat01-200 (a, b, c); *Aaf*Sat11-227 (d, e, f); *Aaf*Sat02-2918 (g, h, i); and *Aaf*Sat03-235 (j, k, l). Bar = 10 μm

Among all the mapped satellites (Table 3), *Aaf*Sat01-200 is an exception to the standard signal distribution, being the only monomer with no signals detected in the Uruguay River – karyomorph B (Fig. 3a). Interestingly, the satDNAs that became dominant in the genome of *A. affinis* karyomorph D colonize the centromeric regions (Fig. 3). For *Aaf*Sat01-200, signals were observed in the centromeric regions of acrocentric pairs 23–26 in Cuiabá River representatives – karyomorph C (Fig. 3b) and in acrocentric pairs 20–26 in the Paraguay River representatives – karyomorph D (Fig. 3c). For *Aaf*Sat11-227, signals were detected in the centromeric region of acrocentric pair 27 in the karyomorph B (Fig. 3d), terminal locations in m/sm pairs 2, 3, and 5, besides on the centromeric region of acrocentric 27 in the karyomorph C (Fig. 3e), and on centromeric regions of acrocentric pairs 20, 21, 22, 23, 26, and 27 in the karyomorph D (Fig. 3f). *Aaf*Sat02-2918 was found to be located in the centromeric region of m/sm pairs 9 and 15, besides acrocentric pair 26 in the karyomorph B (Fig. 3g), on the m/sm pairs 2 and 5 in the karyomorph C (Fig. 3h), and on the m/sm pairs 4, 7, 9, and 10 in the karyomorph D (Fig. 3i). *Aaf*Sat03-235 showed centromeric localization only in m/sm chromosomes, specifically in pairs 5, 12, 15, 21, and 23 in the karyomorph B (Fig. 3j); pairs 12, 15, 16, and 17 in the karyomorph C (Fig. 3k); and pairs 5, 12, 15, 16, and 17 in the karyomorph D (Fig. 3l).

**Table 3:** Number of sites and chromosomal locations of the most abundant satellites in *A. affinis* in metaphases of specimens from the Uruguay, Cuiabá, and Paraguay rivers

satDNA	Uruguay River Karyomorph B 46 m/sm + 4 st + 4a	Cuiabá River Karyomorph C 42 m/sm + 2 st + 10a	Paraguay River Karyomorph D 36 m/sm + 2 st + 16a	Figure
*Aaf*Sat01-200	ND	8a (cen)	14a (cen)	3a, b, c
*Aaf*Sat11-227	2a (cen)*	6 m/sm (t) + 2a (cen)	12a (cen)	3 d, e, f
*Aaf*Sat02-2918	4 m/sm + 2a (cen)	4 m/sm (cen)	8 m/sm (cen)	3 g, h, i
*Aaf*Sat03-235	10 m/sm (cen)	8 m/sm (cen)	10 m/sm (cen)	3j, k, l
*Aaf*Sat04-176	8 m/sm (cen) + 2 st (cen)	4 m/sm (cen) + 2 st (cen)	4 m/sm (cen)	S1a, b, c
*Aaf*Sat05-1323	2 m/sm (t)	2 m/sm (t)	2a (t)	S1d, e, f
*Aaf*Sat06-343	11 m/sm + 1a (cen)*	10 m/sm (cen)	8 m/sm (cen)	S1g, h, i
*Aaf*Sat07-1242	ND	ND	ND	NS
*Aaf*Sat08-434	2 m/sm (cen)	2 m/sm (cen)	2 m/sm (cen)	S2a, b, c
*Aaf*Sat09-33	ND	ND	ND	NS
*Aaf*Sat10-143	2 m/sm (cen)	2 m/sm (cen)	2a (t)	S2d, e, f
*Aaf*Sat12-52	12 m/sm (t) + 2 m/sm (cen)	16 m/sm (t) + 2a (t)	14 m/sm + 6a (t) + 8a (int)	S2g, h, i
*Aaf*Sat13-158	2 m/sm (t)	2 m/sm (t)	1a (prox)	S3a, b, c
*Aaf*Sat14-342	ND	ND	ND	NS
*Aaf*Sat15-814	2 m/sm (t)	2 m/sm (t)	2 m/sm (t)	S3d, e, f
*Aaf*Sat16-1702	2 m/sm (t)	2 m/sm (cen)	2a (int)	S3g, h, i
*Aaf*Sat17-217	2 m/sm (t)	2 m/sm (t)	2a (sub)	S4a, b, c
*Aaf*Sat18-179	6 m/sm (cen)	6 m/sm (cen)	6 m/sm (cen)	S4d, e, f
*Aaf*Sat19-214	2 m/sm (t)	2 m/sm (t)	2 m/sm (t)	S4g, h, i

^*^Heteromorphic pair described by do Nascimento et al. (2018)

cen = centromeric; t = terminal; int = interstitial; prox = proximal; sub = subterminal; ND = not detected; NS = not shown

The satellites *Aaf*Sat04-176 and *Aaf*Sat06-343 exhibited centromeric localization in m/sm and st chromosomes across all three karyomorphs (Table 3, Supplementary Material, Fig. S1). In turn, the *Aaf*Sat05-1323 showed terminal localization in a m/sm pair in karyomorphs B and C, and on an acrocentric pair in karyomorph D (Table 3, Supplementary Material, Fig. S1d, e, f). The *Aaf*Sat08-434 exhibited centromeric localization in m/sm and st chromosomes across all three karyomorphs (Table 3, Supplementary Material, Fig. S2a, b, c). *Aaf*Sat10-143 showed centromeric localization in a m/sm pair in the karyomorphs B and C, and terminal location of an acrocentric pair in the karyomorph D (Table 3, Supplementary Material, Fig. S2d, e, f). In turn, the satellite *Aaf*Sat12-52 was preferentially located in the terminal region of the m/sm chromosomes, but also displayed in a m/sm pair in karyomorph B, an acrocentric pair with terminal location in the karyomorph C, and terminal and interstitial signals on acrocentric chromosomes in the karyomorph D (Table 3, Supplementary Material, Fig. S2g, h, i). The satellites *Aaf*Sat13-158, *Aaf*Sat15-814, and *Aaf*Sat16-1702 showed hybridization signals on a single chromosome pair (Table 3, Supplementary Material, Fig. S3). *Aaf*Sat17-217 showed a terminal location on the m/sm pair in karyomorphs B and C, and in a subterminal location in an acrocentric pair in karyomorph D (Table 3, Supplementary Material, Fig. S4a, b, c). Meanwhile, *Aaf*Sat18-179 showed centromeric localization on the m/sm chromosome pairs, *Aaf*Sat19-214 signals were identified in the terminal location of a m/sm pair (Table 3, Supplementary Material, Fig. S4d-i). Finally, no reliable signals were observed for *Aaf*Sat07-1242, *Aaf*Sat09-33, and *Aaf*Sat14-342 (data not shown).

The annotation of non-B DNA motifs in *A. affinis* satDNAs exhibiting centromeric localization revealed the presence of DR, IR, MR, and STR (Table 4).

**Table 4:** Annotation of the non-B motifs in the satDNA of *A. affinis* (karyomorph D) genome, which showed centromeric localization

Satellite	Non-B motifs
*Aaf*Sat01-200	- 2 DR, 6 IR, 1 MR, and 2 STR 1 IR - 1 IR 3 IR and 1 MR - - -
*Aaf*Sat02-2918	
*Aaf*Sat03-235	
*Aaf*Sat04-176	
*Aaf*Sat06-343	
*Aaf*Sat08-434	
*Aaf*Sat11-227	
*Aaf*Sat12-52	
*Aaf*Sat18-179	

## Discussion

Fish species with satellitome data have shown a high number of satDNA sequences, exceeding several dozen, a quantity higher than in other vertebrate groups (de Silva et al. 2017; Utsunomia et al. 2017, 2019; Crepaldi and Parise-Maltempi 2020; Crepaldi et al. 2021; Deon et al. 2024). In *A. affinis*, we identified 48 satDNAs in the genome, most of which have monomers longer than 100 bp and an A+T content higher than 50%, a characteristic commonly observed in fish satDNAs, as those observed in *Characidium* and *Triportheus* (Serrano-Freitas et al. 2020; Kretschmer et al. 2022).

The satelitome revealed an extensive homogenization of most satDNA families in the *A. affinis* genome, and the low number of superfamily-level satDNA groups aligns well with the satellite dominance alternation model and the main models of repetitive sequence evolution (Dover 1982, 1986; Camacho et al. 2022). The satDNA evolution models explain the homogenization of paralogous copies through molecular mechanisms such as unequal crossing-over, gene conversion, and transposition (Smith 1976; Dover 1986; Plohl et al. 2012). Thus, once a dominant and homogeneous satDNA becomes established in the genome, over time, some monomers may accumulate mutations, losing their identity as members of a satellite family or even a superfamily, reducing sequence similarity to levels below detectability unless new amplification events occur (Camacho et al. 2022). This entire turnover proposition for satDNA explains the sequence diversity in the *A. affinis* genome, which reflects the evolutionary history of the lineage that led to the fixation of monomers lacking superfamily identity. In contrast, others retain shared levels of sequence identity.

Conversely, as phylogenetic distance increases, the retention of orthologous satDNA copies becomes progressively rarer due to their rapid turnover (Camacho et al. 2022). In these cases, according to satDNA library hypothesis, only a residual retention of counterparts of the dominant satDNAs can be found in the genomes of other closely related groups (Fry and Salser 1977). The comparison of the *A. affinis* satellitome with those of other Characiformes revealed low-coverage segments of high identity. This process indicates that the divergence time among lineages, combined with the rapid evolution of satDNA, has driven substantial sequence diversification. Thus, mutations could lead to interspecific diversification of parts of the sequences, and the intragenomic processes of homogenization to which satDNAs are subjected could lead to the expansion of their copy number in a lineage-specific manner, consistent with the rapid evolution of satDNAs among the groups, which has led to satDNA variation (Smith 1976; Fry and Salser 1977; Dover 1986; Plohl et al. 2012).

The maintenance of sequence similarity levels for the replacement of the dominant satDNA is also observed between *Aaf*Sat01-200 and the pPh2004 satellite. *In situ* localization studies have mapped pPh2004 in all *Parodon* species, as well as in karyomorphs A, C, and D of *A. affinis* (Vicente et al. 2003; Schemberger et al. 2011; do Nascimento et al. 2018). However, analysis of *Aaf*Sat01-200 revealed that this satDNA diverged in karyomorphs C and D of *A. affinis* to the point of being considered a satellite distinct from pPh2004 (73.74% sequence homology). The localization of pPh2004 was possible due to the stringent conditions (approximately 70%) applied during the FISH procedure in those studies. Notably, it is worth emphasizing that the satDNAs *Aaf*Sat01-200 and pPh2004 were not localized *in situ* in karyomorph B of *A. affinis* (do Nascimento et al. 2018; present study). This observation opens the possibility of investigating counterparts of the pPh2004 unit that may have diversified into a different satellite in this karyomorph. This hypothesis is further supported by the occurrence of *Aaf*Sat01-200 in the centromeric regions of the acrocentric chromosomes that diversified in karyomorphs C and D of *A. affinis*, while the number of acrocentrics in karyomorph B remains low.

**Fig. 4:**
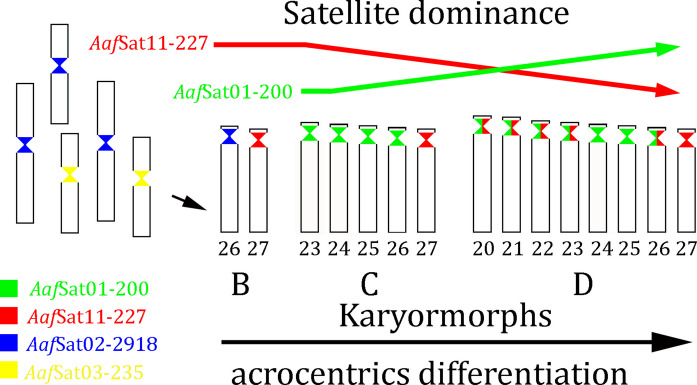
Proposals of centromere repositioning in acrocentric chromosomes of *A. affinis* karyomorph D. Within *A. affinis* karyomorph D, the satellite DNA families *Aaf*Sat01-200 and *Aaf*Sat11-227, as well as *Aaf*Sat02-2918 and *Aaf*Sat03-235, have experienced a reorganization of their genomic dominance. *Aaf*Sat02-2918 and *Aaf*Sat03-235 remain preferentially centromeric in the m/sm chromosomes, whereas *Aaf*Sat01-200 is exclusively centromeric in acrocentrics of both karyomorphs C and D. *Aaf*Sat11-227, however, is found in terminal regions of the m/sm chromosomes in karyomorph C and at the centromeric region of acrocentric pair 27 in all three karyomorphs. The absence of *Aaf*Sat11-227 on pairs 23–26 in karyomorph C indicates that inversions cannot account for the origin of these acrocentric chromosomes. Conversely, these patterns are consistent with centromere repositioning, in which an ancestral centromere is progressively lost as a new one emerges on the same chromosome, typically revealed by the absence of the original centromeric repeats at the new locus. The shift in dominance toward the AT-rich satellite *Aaf*Sat01-200 in karyomorph D, reflected by its increased copy number and low sequence divergence, supports the hypothesis that this satellite may have acquired a centromeric role in the acrocentric chromosomes

The differentiation of acrocentric chromosomes in *A. affinis*, evidenced by shifts in the m/sm and st/a ratios across populations in the Lower Paraná River basin, has been proposed to result from the influence of repetitive DNA sequences. do Nascimento et al. (2018) suggested that the expansion of acrocentric chromosomes in this species was driven by structural rearrangements, particularly pericentric inversions or centromeric repositioning. Our data showed that satellites *Aaf*Sat01-200 and *Aaf*Sat11-227, as well as *Aaf*Sat02-2918 and *Aaf*Sat03-235, have undergone shifts in genome dominance in the *A. affinis* karyomorph D. While *Aaf*Sat02-2918 and *Aaf*Sat03-235 are preferentially centromeric in m/sm chromosomes, the *Aaf*Sat01-200 is exclusively centromeric in acrocentrics in the karyomorphs C and D, opening a perspective on its role in centromeric repositioning (Fig. 4). Centromeric repositioning refers to the emergence of a new centromere, with significant implications for chromosome stability and function (Montefalcone et al. 1999; Amor et al. 2004; Schubert 2018). Schubert (2018) proposed that the gradual loss of an ancestral centromere, accompanied by the emergence of a new one on the same chromosome, can be inferred from the absence at the new locus of the repetitive sequences characteristic of the original centromere. Thus, concomitant with the stabilization in the copy number of *Aaf*Sat11-227, the satellite *Aaf*Sat01-200 underwent a marked expansion in the genome of *A. affinis* karyomorph D, characterized by a higher number of copies with fewer accumulated divergences. According to this evidence, the satellite *Aaf*Sat01-200 could have acquired a centromeric function in the acrocentric chromosomes, rapidly becoming the dominant one due to satDNA homogenization mechanisms in *A. affinis* karyomorph D, a process also recognized in other organisms (Dover 1982; Camacho et al. 2022).

In addition, whereas *Aaf*Sat01-200 has preferential loci in the centromeric regions of acrocentrics, the *Aaf*Sat02-2918 and AafSat03-235 satellites are widely distributed across the centromeric regions of m/sm chromosomes. The nucleotide divergence graph shows that *Aaf*Sat02-2918 is in the process of increasing the number of copies with low or no divergence, while *Aaf*Sat03-235 shows a greater number of copies with higher divergence values. By overlapping the divergence graphs of satellites *Aaf*Sat01-200, *Aaf*Sat11-227, *Aaf*Sat02-2918, and *Aaf*Sat03-235, it is possible to suppose that the reorganization of the centromeric regions in acrocentric chromosomes was accompanied by a restructuring of the centromeres in some m/sm chromosomes. In addition, these two satellites do not account for the centromeric organization of all m/sm and st chromosomes in karyomorph D. Although centromeres share the standard function of ensuring accurate chromosome segregation, they differ remarkably among species in both sequence composition and structural organization (Enriquez and Nechemia‑Arbely 2025).

In some equid lineages, Robertsonian rearrangements have generated chromosomes with satellite-free centromeres (Cappelletti et al. 2022, 2025). In several of these cases, satellite-free neocentromeres have been proposed to undergo a subsequent “satellitization” process, in which newly recruited satellite repeats become incorporated into the developing centromeric domain (Nergadze et al. 2018). There is currently no evidence that *A. affinis* experienced satellite-free neocentromeres during lineage differentiation. Nonetheless, a process analogous to “satellitization” can be inferred from the origin and expansion of *Aaf*Sat01-200, an AT-rich satellite DNA localized to acrocentric centromeres, which likely contributed to the structural “maturation” of these centromeres over evolutionary time. This diversity underscores the dynamic nature of centromere evolution and suggests that different species have adopted distinct strategies to maintain centromere stability and function.

Another line of evidence comes from studies in grasshoppers, where no conserved functional motifs have been identified in centromeric monomers (Camacho et al. 2022). As proposed for grasshoppers, *A. affinis* may also rely on a diverse set of satellite sequences to support centromeric function. This possibility is particularly evident in the m/sm chromosomes of karyomorph D, whose different centromeres appear to be colonized by distinct satellites, including *Aaf*Sat02-2918, *Aaf*Sat03-235, *Aaf*Sat04-176, *Aaf*Sat06-343, *Aaf*Sat08-434, *Aaf*Sat12-52, and *Aaf*Sat18-179. Except for *Aaf*Sat02-2918 and *Aaf*Sat03-235, which show higher copy numbers and greater variability, these satellites are relatively more stable across the three karyomorphs, suggesting a longer-term presence in the lineage. In species lacking a characteristic centromeric sequence motif, it has been proposed that inverted repeats within satDNA can promote the formation of thermodynamically stable non-B DNA structures that serve as targets for centromeric protein recruitment (Koch 2000; Hall et al. 2003; Luchetti et al. 2003; Camacho et al. 2022). Given that cruciform structures within satellite monomers can generate such non-B conformations and facilitate centromere assembly (Kasinathan and Henikoff 2018; Talbert and Henikoff 2025), the non-B DNA motifs identified in *Aaf*Sat02-2918, *Aaf*Sat03-235, *Aaf*Sat06-343, and *Aaf*Sat08-434 of *A. affinis* support the hypothesis that these sequences may contribute to centromere function.

On the other hand, it is known that new centromeres can arise from novel DNA sequences. In such cases, centromere emergence may be accompanied, or even facilitated, by the rapid expansion of novel satDNA, as newly expanded AT-rich repeat arrays can reshape the local chromatin landscape and promote CENP-A deposition (Murillo-Pineda and Jansen 2020; Murillo-Pineda et al. 2021). In our data, we observed that the satDNAs located in the centromeres of acrocentric chromosomes in *A. affinis* lack detectable non-B DNA conformations. However, *Aaf*Sat01-200 represents a recently amplified satellite family forming long AT-rich arrays within these centromeres. This pattern suggests that *Aaf*Sat01-200 likely emerged in the *A. affinis* lineage and became associated with centromere function in the acrocentric chromosomes. This interpretation is further supported by the absence of evidence for pericentric inversions and by the observation that satDNAs with centromeric localization in m/sm chromosomes are not present at the centromeres of the acrocentrics. This view aligns with recent proposals that centromeres comprise multiple, functionally distinct chromatin domains, implying that the expansion of novel AT-rich satDNA during neocentromere formation may contribute not only to CENP-A recruitment but also to shaping the broader multilayered centromeric architecture (Corless et al. 2025). Functional assays such as CENP-A ChIP enrichment, combined with immuno-FISH colocalization and targeted deletion or ectopic array recruitment experiments, could directly test whether *Aaf*Sat01-200 plays a centromeric role.

## Conclusion

The satellitome data generated for *A. affinis* from the Paraguay River (karyomorph D) in this study, along with the chromosomal localization of the most abundant sequences, demonstrated that *Aaf*Sat01-200 rapidly became the dominant sequence present in the acrocentric chromosomes of karyomorphs C and D. Reinforced by the lack of a consistent chromosomal signature to support pericentric inversions, the data demonstrated the involvement of the *Aaf*Sat01-200 in centromeric repositioning, which could explain the increasing number of acrocentric chromosomes in these populations. Additionally, the study highlights that numerous *A. affinis* satellite monomers adopting non-B DNA conformations may participate in centromere organization, and it further reveals a clear distinction between the monomers located on m/sm chromosomes and those found on acrocentric chromosomes.

## Supplementary Information

Below is the link to the electronic supplementary material.

Supplementary file1 (PDF 580 KB)

## Data Availability

All data supporting the findings of this study are included in this published article and its supplementary information files.

## Abbreviations

2n: Diploid chromosome number
a: Acrocentric
APR: A-phased repeats
bp: Base pair
BLASTn: Basic Local Alignment Search Tool for nucleotides
DAPI: 4,6-Diamidino-2-phenylindole
DR: Direct repeats
DSBs: Double-strand Breaks
FISH: Fluorescence* in situ* Hybridization
IR: Inverted repeats
m: Metacentric
MR: Mirror repeats
K2P: Kimura-2-parameter
NAHR: Non-allelic Homologous Recombination
NGS: Next-Generation Sequencing
PCR: Polymerase Chain Reaction
pPh2004: Satellite DNA from *Parodon hilarii*
rDNA: Ribosomal DNA
RUL: Repeat Unit Lengths
satDNA: Satellite DNA
SF: Superfamily (of satDNA)
sm: Submetacentric
SSC: Saline-Sodium Citrate
st: Subtelocentric
STR: Short tandem repeats
TAREAN: Tandem Repeat Analyzer
TEs: Transposable elements

## References

[CR1] Altschul SF, Gish W, Miller W, Myers EW, Lipman DJ (1990) Basic local alignment search tool. J Mol Biol 215:403–410. 10.1016/S0022-2836(05)80360-2 2231712 10.1016/S0022-2836(05)80360-2

[CR2] Amor DJ, Bentley K, Ryan J, Perry J, Wong L, Slater H, Choo KHA (2004) Human centromere repositioning bin progress. Proc Natl Acad Sci U S A 101:6542–6547. 10.1073/pnas.0308637101 15084747 10.1073/pnas.0308637101 PMC404081

[CR3] Azambuja M, Schemberger MO, Nogaroto V, Moreira-Filho O, Martins C, Vicari MR (2022) Major and minor U small nuclear RNAs genes characterization in a neotropical fish genome: Chromosomal remodeling and repeat units dispersion in Parodontidae. Gene 826:146459. 10.1016/j.gene.2022.146459 35358649 10.1016/j.gene.2022.146459

[CR4] Azambuja M, Nogaroto V, Moreira-Filho O, Vicari MR (2023) U2 and U4 snDNA comparative chromosomal mapping in the neotropical fish genera *Apareiodon* and *Parodon* (Characiformes: Parodontidae). Zebrafish 20:5. 10.1089/zeb.2023.0025 10.1089/zeb.2023.0025 37797225

[CR5] Bellafronte E, Schemberger MO, Moreira-Filho O, Almeida MC, Artoni RF, Margarido VP, Vicari MR (2011) Chromosomal markers in Parodontidae: an analysis of new and reviewed data with phylogenetic inferences. Rev Fish Biol Fish 21:559–570. 10.1007/s11160-010-9177-3

[CR6] Bertollo LAC, Cioffi MB, Moreira-Filho O (2015) Direct chromosome preparation from freshwater teleost fishes. In: Ozouf-Costaz C, Pisano E, Foresti F, Almeida Toledo LF (eds) Fish cytogenetic techniques (Chondrichthyans and Teleosts), 1st edn. CRC Press, Boca Raton, pp 21–26. 10.1201/b18534-4

[CR7] Biscotti MA, Olmo E, Heslop-Harrison JS (2015) Repetitive DNA in eukaryotic genomes. Chromosome Res 23:415–420. 10.1007/s10577-015-9499-z 26514350 10.1007/s10577-015-9499-z

[CR8] Camacho JPM, Cabrero J, López-León MD, Martín-Peciña M, Perfectti F, Garrido-Ramos MA, Ruiz-Ruano FJ (2022) Satellitome comparison of two oedipodine grasshoppers highlights the contingent nature of satellite DNA evolution. BMC Biol 20:36. 10.1186/s12915-021-01216-9 35130900 10.1186/s12915-021-01216-9 PMC8822648

[CR9] Cappelletti E, Piras FM, Sola L, Santagostino M, Abdelgadir WA, Raimondi E, Lescai F, Nergadze SG, Giulotto E (2022) Robertsonian fusion and centromere repositioning contributed to the formation of satellite-free centromeres during the evolution of zebras. Mol Biol Evol 39:msac162. 10.1093/molbev/msac162 35881460 10.1093/molbev/msac162 PMC9356731

[CR10] Cappelletti E, Piras FM, Biundo M, Bellone RR, Finno CJ, Kalbfleisch TS, Petersen JL, Nergadze SG, Giulotto E (2025) CENP-A and centromere evolution in equids. Chromosom Res 33:13. 10.1007/s10577-025-09773-3 10.1007/s10577-025-09773-3 PMC12208984 40586953

[CR11] Cech TR, Steitz JA (2014) The noncoding RNA revolution – trashing old rules to forge new ones. Cell 157:77–94. 10.1016/j.cell.2014.03.008 24679528 10.1016/j.cell.2014.03.008

[CR12] Cer RZ, Donohue DE, Mudunuri US, Temiz NA, Loss MA, Starner NJ, Halusa GN, Volfovsky N, Yi M, Luke BT, Bacolla A, Collins JR, Stephens RM (2013) Non-B DB v2.0: a database of predicted non-B DNA-forming motifs and its associated tools. Nucleic Acids Res 41:D94–D100. 10.1093/nar/gks955 23125372 10.1093/nar/gks955 PMC3531222

[CR13] Cesari M, Luchetti A, Passamonti M, Scali V, Mantovani B (2003) Polymerase chain reaction amplification of the Bag320 satellite family reveals the ancestral library and past gene conversion events in *Bacillus rossius* (Insecta Phasmatodea). Gene 312:289–295. 10.1016/S0378-1119(03)00625-5 12909366 10.1016/s0378-1119(03)00625-5

[CR14] Corless S, Thangavel G, Erhardt S (2025) Revisiting the question: when is a centromere not a kinetochore? Chromosom Res 33:23. 10.1007/s10577-025-09782-2 10.1007/s10577-025-09782-2 PMC12553604 41137946

[CR15] Crepaldi C, Parise-Maltempi PP (2020) Heteromorphic sex chromosomes and their DNA content in fish: an insight through satellite DNA accumulation in *Megaleporinus elongatus*. Cytogenet Genome Res 160:38–46. 10.1159/000506265 32092756 10.1159/000506265

[CR16] Crepaldi C, Martí E, Gonçalves ÉM, Martí DA, Parise-Maltempi PP (2021) Genomic differences between the sexes in a fish species seen through satellite DNAs. Front Genet 12:728670. 10.3389/fgene.2021.728670 34659353 10.3389/fgene.2021.728670 PMC8514694

[CR17] de Oliveira FS, Azambuja M, Schemberger MO, Nascimento VD, Oliveira JIN, Wolf IR, Nogaroto V, Martins C, Vicari MR (2024) Characterization of *hAT* DNA transposon superfamily in the genome of Neotropical fish *Apareiodon* sp. Mol Genet Genomics 299:96. 10.1007/s00438-024-02190-x 39382723 10.1007/s00438-024-02190-x

[CR18] de Oliveira FS, Brann T, Wolf IR, Nogaroto V, Martins C, Protasio AV, Vicari MR (2025) The landscape of transposable elements distribution in the genome of Neotropical fish *Apareiodon* sp. (Characiformes: Parodontidae). Chromosome Res 33:6. 10.1007/s10577-025-09765-3 40186682 10.1007/s10577-025-09765-3

[CR19] de Silva DMZA, Utsunomia R, Ruiz-Ruano FJ, Daniel SN, Porto-Foresti F, Hashimoto DT, Oliveira C, Camacho JPM, Foresti F (2017) High-throughput analysis unveils a highly shared satellite DNA library among three species of fish genus *Astyanax*. Sci Rep 7:12726. 10.1038/s41598-017-12939-7 29018237 10.1038/s41598-017-12939-7 PMC5635008

[CR20] del Bosque MEQ, López-Flores I, Suárez-Santiago VN, Garrido-Ramos MA (2014) Satellite-DNA diversification and the evolution of major lineages in Cardueae (Carduoideae Asteraceae). J Plant Res 127:575–583. 10.1007/s10265-014-0648-9 25030895 10.1007/s10265-014-0648-9

[CR21] Deon GA, dos Santos RZ, Sassi FMC, Moreira-Filho O, Vicari MR, Porto-Foresti F, Utsunomia R, Cioffi MB (2024) The role of satellite DNAs in the chromosomal rearrangements and the evolution of the rare XY_1_Y_2_ sex system in *Harttia* (Siluriformes: Loricariidae). J Hered 115:541–551. 10.1093/JHERED/ESAE028 38757192 10.1093/jhered/esae028

[CR22] do Nascimento VD, Coelho KA, Nogaroto V, de Almeida RB, Ziemniczak K, Centofante L, Pavanelli CS, Torres RA, Moreira-Filho O, Vicari MR (2018) Do multiple karyomorphs and population genetics of freshwater darter characines (*Apareiodon affinis*) indicate chromosomal speciation? Zool Anz 272:93–103. 10.1016/j.jcz.2017.12.006

[CR23] Dover G (1982) Molecular drive: a cohesive mode of species evolution. Nature 299:111–117. 10.1038/299111a0 7110332 10.1038/299111a0

[CR24] Dover G (1986) Molecular drive in multigene families: how biological novelties arise, spread and are assimilated. Trends Genet 2:159–165. 10.1016/0168-9525(86)90211-8

[CR25] Enriquez A, Nechemia-Arbely Y (2025) The dynamic centromere. Chromosome Res 33:22. 10.1007/s10577-025-09779-x 41099875 10.1007/s10577-025-09779-x PMC12532704

[CR26] Farré M, Bosch M, López-Giráldez F, Ponsà M, Ruiz-Herrera A (2011) Assessing the role of tandem repeats in shaping the genomic architecture of great apes. PLoS One 6:27239. 10.1371/journal.pone.0027239 10.1371/journal.pone.0027239 PMC3208591 22076140

[CR27] Fricke R, Eschmeyer WN, Van-der-Laan R (2025) Eschmeyer’s catalog of fishes: genera, species, references. California Academy of Science, San Francisco. https://researcharchive.calacademy.org/research/ichthyology/catalog/fishcatmain.asp Accessed 26 October 2025

[CR28] Fry K, Salser W (1977) Nucleotide sequence of HS-α satellite DNA from kangaroo rat *Dipodomys ordii* and characterization of similar sequences in other rodents. Cell 12:1059–1084. 10.1016/0092-8674(77)90170-2 10.1016/0092-8674(77)90170-2 597857

[CR29] Garrido-Ramos MA (2015) Satellite DNA in plants: more than just rubbish. Cytogenet Genome Res 146:153–170. 10.1159/000437008 26202574 10.1159/000437008

[CR30] Garrido-Ramos MA (2017) Satellite DNA: an evolving topic. Genes 8:230. 10.3390/genes8090230 28926993 10.3390/genes8090230 PMC5615363

[CR31] Glugoski L, Giuliano-Caetano L, Moreira-Filho O, Vicari MR, Nogaroto V (2018) Co-located hAT transposable element and 5S rDNA in an interstitial telomeric sequence suggest the formation of Robertsonian fusion in armored catfish. Gene 650:49–54. 10.1016/j.gene.2018.01.099 29408629 10.1016/j.gene.2018.01.099

[CR32] Glugoski L, Nogaroto V, Deon GA, Azambuja M, Moreira-Filho O, Vicari MR (2022) Enriched tandem repeats in chromosomal fusion points of *Rineloricaria latirostris* (Boulenger, 1900) (Siluriformes: Loricariidae). Genome 65:479–489. 10.1139/gen-2022-0043 35939838 10.1139/gen-2022-0043

[CR33] Goes CAG, dos Santos RZ, Aguiar WRC, Alves DCV, Silva DMZA, Foresti F, Oliveira C, Utsunomia R, Porto-Foresti F (2022) Revealing the satellite DNA history in *Psalidodon* and *Astyanax* characid fish by comparative satellitomics. Front Genet 13:884072. 10.3389/fgene.2022.884072 35801083 10.3389/fgene.2022.884072 PMC9253505

[CR34] Goes CAG, Santos N, Rodrigues PHM, Stornioli JHF, da Silva AB, dos Santos RZ, Vidal JAD, Silva DMZA, Artoni RF, Foresti F, Hashimoto DT, Porto-Foresti F, Utsunomia R (2023) The satellite DNA catalogues of two Serrasalmidae (Teleostei, Characiformes): conservation of general satDNA features over 30 million years. Genes 14:91. 10.3390/genes14010091 10.3390/genes14010091 PMC9859320 36672835

[CR35] Hall SE, Kettler G, Preuss D (2003) Centromere satellites from *Arabidopsis* populations: maintenance of conserved and variable domains. Genome Res 13:195–205. 10.1101/gr.593403 12566397 10.1101/gr.593403 PMC420371

[CR36] Hartley G, O’Neill RJ (2019) Centromere repeats: hidden gems of the genome. Genes 10:223. 10.3390/GENES10030223 30884847 10.3390/genes10030223 PMC6471113

[CR37] Kasinathan S, Henikoff S (2018) Non-B-form DNA is enriched at centromeres. Mol Biol Evol 35:949–962. 10.1093/molbev/msy010 29365169 10.1093/molbev/msy010 PMC5889037

[CR38] Koch J (2000) Neocentromeres and alpha satellite: a proposed structural code for functional human centromere DNA. Hum Mol Genet 9:149–154. 10.1093/hmg/9.2.149 10607825 10.1093/hmg/9.2.149

[CR39] Kohany O, Gentles AJ, Hankus L, Jurka J (2006) Annotation, submission and screening of repetitive elements in Repbase: RepbaseSubmitter and censor. BMC Bioinformatics 7:474. 10.1186/1471-2105-7-474 17064419 10.1186/1471-2105-7-474 PMC1634758

[CR40] Koukalova B, Moraes AP, Renny-Byfield S, Matyasek R, Leitch AR, Kovarik A (2010) Fall and rise of satellite repeats in allopolyploids of *Nicotiana* over c. 5 million years. New Phytol 186:148–160. 10.1111/J.1469-8137.2009.03101.X 19968801 10.1111/j.1469-8137.2009.03101.x

[CR41] Kretschmer R, Goes CAG, Bertollo LAC, Ezaz T, Porto-Foresti F, Toma GA, Utsunomia R, Cioffi MB (2022) Satellitome analysis illuminates the evolution of ZW sex chromosomes of Triportheidae fishes (Teleostei: Characiformes). Chromosoma 131:29–45. 10.1007/s00412-022-00768-1 35099570 10.1007/s00412-022-00768-1

[CR42] Kuhn GCS (2015) Satellite DNA transcripts have diverse biological roles in *Drosophila*. Heredity 115:1–2. 10.1038/hdy.2015.12 25806543 10.1038/hdy.2015.12 PMC4815497

[CR43] Levan A, Fredga K, Sandberg AA (1964) Nomenclature for centromeric position on chromosomes. Hereditas 52:201–220. 10.1111/J.1601-5223.1964.TB01953.X

[CR44] Lower SS, McGurk MP, Clark AG, Barbash DA (2018) Satellite DNA evolution: old ideas, new approaches. Curr Opin Genet Dev 49:70–78. 10.1016/j.gde.2018.03.003 29579574 10.1016/j.gde.2018.03.003 PMC5975084

[CR45] Luchetti A, Cesari M, Carrara G, Cavicchi S, Passamonti M, Scali V, Mantovani B (2003) Unisexuality and molecular drive: Bag320 sequence diversity in *Bacillus* taxa (Insecta Phasmatodea). J Mol Evol 56:587–596. 10.1007/s00239-002-2427-9 12698295 10.1007/s00239-002-2427-9

[CR46] Meštrović N, Mravinac B, Plohl M, Ugarković D, Bruvo-Madarić B (2006) Preliminary phylogeny of *Tribolium* beetles (Coleoptera: Tenebrionidae) resolved by combined analysis of mitochondrial genes. Eur J Entomol 103:709–715. 10.14411/eje.2006.094

[CR47] Meštrović N, Plohl M, Mravinac B, Ugarković D (1998) Evolution of satellite DNAs from the genus *Palorus*-experimental evidence for the library hypothesis. Mol Biol Evol 15:1062–1068. 10.1093/OXFORDJOURNALS.MOLBEV.A026005 9718733 10.1093/oxfordjournals.molbev.a026005

[CR48] Montefalcone G, Tempesta S, Rocchi M, Archidiacono N (1999) Centromere repositioning. Genome Res 9:1184–1188. 10.1101/gr.9.12.1184 10613840 10.1101/gr.9.12.1184 PMC311001

[CR49] Murillo-Pineda M, Jansen LET (2020) Genetics, epigenetics and back again: lessons learned from neocentromeres. Exp Cell Res 389:111909. 10.1016/j.yexcr.2020.111909 32068000 10.1016/j.yexcr.2020.111909

[CR50] Murillo-Pineda M, Valente LP, Dumont M, Mata JF, Fachinetti D, Jansen LE (2021) Induction of spontaneous human neocentromere formation and long-term maturation. J Cell Biol 220:e202007210. 10.1083/jcb.202007210 33443568 10.1083/jcb.202007210 PMC7812830

[CR51] Nei M, Rooney AP (2005) Concerted and birth-and-death evolution of multigene families. Annu Rev Genet 39:121–152. 10.1146/annurev.genet.39.073003.112240 16285855 10.1146/annurev.genet.39.073003.112240 PMC1464479

[CR52] Nergadze SG, Piras FM, Gamba R, Corbo M, Cerutti F, McCarter JGW, Cappelletti E, Gozzo F, Harman RM, Antczak DF, Miller D, Scharfe M, Pavesi G, Raimondi E, Sullivan KF, Giulotto E (2018) Birth, evolution, and transmission of satellite-free mammalian centromeric domains. Genome Res 28:789–799. 10.1101/gr.231159.117 29712753 10.1101/gr.231159.117 PMC5991519

[CR53] Nirchio M, Masache MC, Paim FG, Cioffi MB, Filho OM, Barriga R, Oliveira C, Rossi AR (2021) Chromosome analysis in *Saccodon wagneri* (Characiformes) and insights into the karyotype evolution of Parodontidae. Neotrop Ichthyol 19:200103. 10.1590/1982-0224-2020-0103

[CR54] Novák P, Neumann P, Macas J (2010) Graph-based clustering and characterization of repetitive sequences in next-generation sequencing data. BMC Bioinformatics 11:378. 10.1186/1471-2105-11-378 20633259 10.1186/1471-2105-11-378 PMC2912890

[CR55] Novák P, Neumann P, Pech J, Steinhaisl J, MacAs J (2013) Repeatexplorer: a galaxy-based web server for genome-wide characterization of eukaryotic repetitive elements from next-generation sequence reads. Bioinformatics 29:792–793. 10.1093/BIOINFORMATICS/BTT054 23376349 10.1093/bioinformatics/btt054

[CR56] Novák P, Robledillo LÁ, Koblížková A, Vrbová I, Neumann P, Macas J (2017) TAREAN: a computational tool for identification and characterization of satellite DNA from unassembled short reads. Nucleic Acids Res 45:111. 10.1093/nar/gkx257 10.1093/nar/gkx257 PMC5499541 28402514

[CR57] Novák P, Neumann P, Macas J (2020) Global analysis of repetitive DNA from unassembled sequence reads using RepeatExplorer2. Nat Protoc 15:3745–3776. 10.1038/s41596-020-0400-y 33097925 10.1038/s41596-020-0400-y

[CR58] O’Neill RJ, Eldridge MDB, Metcalfe CJ (2004) Centromere dynamics and chromosome evolution in marsupials. J Hered 95:375–381. 10.1093/JHERED/ESH063 15388765 10.1093/jhered/esh063

[CR59] Pavanelli CS (2003) Family Parodontidae (Parodonts). In: Reis RE, Sven OK, Ferraris Junior CJ (eds) Check List of the Freshwaters of South and Central América, 1st edn. EDIPUCRS, Porto Alegre, pp 46–50

[CR60] Peschansky VJ, Wahlestedt C (2014) Non-conding RNAs as direct and indirect modulators of epigenetic regulation. Epigenetics 9:3–12. 10.4161/epi.27473 24739571 10.4161/epi.27473 PMC3928183

[CR61] Pinkel D, Straume T, Gray JW (1986) Cytogenetic analysis using quantitative, high-sensitivity, fluorescence hybridization. Proc Natl Acad Sci U S A 83:2934–2938. 10.1073/PNAS.83.9.2934 3458254 10.1073/pnas.83.9.2934 PMC323421

[CR62] Plohl M, Meštrović N, Mravinac B (2012) Satellite DNA evolution. Genome Dyn 7:126–152. 10.1159/000337122 22759817 10.1159/000337122

[CR63] Richard GF, Kerrest A, Dujon B (2008) Comparative genomics and molecular dynamics of DNA repeats in eukaryotes. Microbiol Mol Biol Rev 72:686–727. 10.1128/mmbr.00011-08 19052325 10.1128/MMBR.00011-08 PMC2593564

[CR64] Rocchi M, Archidiacono N, Schempp W, Capozzi O, Stanyon R (2012) Centromere repositioning in mammals. Heredity 108:59–67. 10.1038/hdy.2011.101 22045381 10.1038/hdy.2011.101 PMC3238114

[CR65] Ruiz-Ruano FJ, López-León MD, Cabrero J, Camacho JPM (2016) High-throughput analysis of the satellitome illuminates satellite DNA evolution. Sci Rep 6:28333. 10.1038/srep28333 27385065 10.1038/srep28333 PMC4935994

[CR66] Samoluk SS, Robledo G, Bertioli D, Seijo JG (2017) Evolutionary dynamics of an at-rich satellite DNA and its contribution to karyotype differentiation in wild diploid *Arachis* species. Mol Genet Genomics 292:283–296. 10.1007/S00438-016-1271-3 27838847 10.1007/s00438-016-1271-3

[CR67] Šatović-Vukšić E, Plohl M (2023) Satellite DNAs-from localized to highly dispersed genome components. Genes 14:742. 10.3390/genes14030742 36981013 10.3390/genes14030742 PMC10048060

[CR68] Schemberger MO, Bellafronte E, Nogaroto V, Almeida MC, Schühli GS, Artoni RF, Moreira-Filho O, Vicari MR (2011) Differentiation of repetitive DNA sites and sex chromosome systems reveal closely related group in Parodontidae (Actinopterygii: Characiformes). Genetica 139:1499–1508. 10.1007/s10709-012-9649-6 22527690 10.1007/s10709-012-9649-6

[CR69] Schubert I (2018) What is behind “centromere repositioning”? Chromosoma 127:229–234. 10.1007/s00412-018-0672-y 29705818 10.1007/s00412-018-0672-y

[CR70] Serrano-Freitas ÉA, Silva DMZA, Ruiz-Ruano FJ, Utsunomia R, Araya-Jaime C, Oliveira C, Camacho JPM, Foresti F (2020) Satellite DNA content of B chromosomes in the characoid fish *Characidium gomesi* supports their origin from sex chromosomes. Mol Genet Genomics 295:195–207. 10.1007/s00438-019-01615-2 31624915 10.1007/s00438-019-01615-2

[CR71] Slijepcevic P (2016) Mechanisms of the evolutionary chromosome plasticity: integrating the ‘centromere-from-telomere’ hypothesis with telomere lenght regulation. Cytogenet Genome Res 148:268–278. 10.1159/000447415 27398800 10.1159/000447415

[CR72] Smit AFA, Hubley R, Green P (2013–2015) RepeatMasker Open¬4.0. http:// www. repeatmasker.org. Accessed 15 Nov 2024

[CR73] Smith GP (1976) Evolution of repeated DNA sequences by unequal crossover: DNA whose sequence is not maintained by selection will develop periodicities as a result of random crossover. Science 191:528–535. 10.1126/science.1251186 1251186 10.1126/science.1251186

[CR74] Storer J, Hubley R, Rosen J, Wheeler TJ, Smit AF (2021) The Dfam community resource of transposable element families, sequence models, and genome annotations. Mob DNA 12:2. 10.1186/s13100-020-00230-y 33436076 10.1186/s13100-020-00230-y PMC7805219

[CR75] Talbert PB, Henikoff S (2025) Centromeres drive and take a break. Chromosome Res 33:17. 10.1007/s10577-025-09777-z 40759764 10.1007/s10577-025-09777-z PMC12321929

[CR76] Traldi JB, Vicari MR, Martinez JF, Blanco DR, Lui RL, Azambuja M, Almeida RB, Malimpensa GC, Costa-Silva GJ, Oliveira C, Pavanelli CS, Moreira-Filho O (2020) Recent *Apareiodon* species evolutionary divergence (Characiformes: Parodontidae) evidenced by chromosomal and molecular inference. Zool Anz 289:166–176. 10.1016/j.jcz.2020.10.010

[CR77] Untergasser A, Nijveen H, Rao X, Bisseling T, Geurts R, Leunissen JAM (2007) Primer3plus, an enhanced web interface to Primer3. Nucleic Acids Res 35:W71–W74. 10.1093/NAR/GKM306 17485472 10.1093/nar/gkm306 PMC1933133

[CR78] Utsunomia R, Ruiz-Ruano FJ, Silva DMZA, Serrano ÉA, Rosa IF, Scudeler PES, Hashimoto DT, Oliveira C, Camacho JPM, Foresti F (2017) A glimpse into the satellite DNA library in characidae fish (Teleostei, Characiformes). Front Genet 8:103. 10.3389/fgene.2017.00103 28855916 10.3389/fgene.2017.00103 PMC5557728

[CR79] Utsunomia R, Silva DMZA, Ruiz-Ruano FJ, Goes CAG, Melo S, Ramos LP, Oliveira C, Porto-Foresti F, Foresti F, Hashimoto DT (2019) Satellitome landscape analysis of *Megaleporinus macrocephalus* (Teleostei, Anostomidae) reveals intense accumulation of satellite sequences on the heteromorphic sex chromosome. Sci Rep 9:5856. 10.1038/s41598-019-42383-8 30971780 10.1038/s41598-019-42383-8 PMC6458115

[CR80] Vicari MR, Bruschi DP, Cabral-de-Mello DC, Nogaroto V (2022) Telomere organization and the interstitial telomeric sites involvement in insects and vertebrates chromosome evolution. Genet Mol Biol 45:20220071. 10.1590/1678-4685-GMB-2022-0071 10.1590/1678-4685-GMB-2022-0071 PMC9693754 36394537

[CR81] Vicente VE, Bertollo LAC, Valentini SR, Moreira-Filho O (2003) Origin and differentiation of a sex chromosome system in *Parodon hilarii* (Pisces, Parodontidae). Satellite DNA G- and C- banding. Genetica 119:115–120. 10.1023/A:1026082904672 14620951 10.1023/a:1026082904672

[CR82] Wells JN, Feschotte C (2020) A field guide to eukaryotic transposable elements. Annu Rev Genet 54:539–561. 10.1146/annurev-genet-040620-022145 32955944 10.1146/annurev-genet-040620-022145 PMC8293684

[CR83] Wicker T, Sabot F, Hua-Van A, Bennetzen JL, Capy P, Chalhoub B, Flavell A, Leroy P, Morgante M, Panaud O, Paux E, SanMiguel P, Schulman AH (2007) A unified classification system for eukaryotic transposable elements. Nat Rev Genet 8:973–982. 10.1038/nrg2165 17984973 10.1038/nrg2165

[CR84] Wolf IR, Schemberger MO, Azambuja M, de Oliveira FS, Nogaroto V, Valente GT, Martins C, Vicari MR (2024) The long-read assembly of *Apareiodon* sp., a neotropical fish with a ZZ/ZW sex chromosome system. Genet Mol Biol 47(4):e20240098. 10.1590/1678-4685-GMB-2024-0098 10.1590/1678-4685-GMB-2024-0098 PMC11468460 39392722

[CR85] Zhang Y, Guo L, Gonzales PK, Gendron TF, Wu Y, Jansen-West K, O’Raw AD, Pickles SR, Prudencio M, Carlomagno Y, Gachechiladze MA, Ludwig C, Tian R, Chew J, Deture M, Lin W, Tonga J, Daughrity LM, Yue M, Song Y, Andersen JW, Castanedes-Casey M, Kurti A, Datta A, Antognetti G, McCampbell A, Rademakers R, Oskarsson B, Dickson DW, Kampmann M, Ward ME, Fryer JD, Link CD, Shorter J, Petrucelli L (2019) Heterochromatin anomalies and double-stranded RNA accumulation underlie C9orf72 poly(PR) toxicity. Science 363:eaav2606. 10.1126/science.aav2606 30765536 10.1126/science.aav2606 PMC6524780

