# Supplementary material for: Emergence of satellite DNAs suggests centromeric repositioning as a driver of karyotypic variation of the freshwater darter characines (Apareiodon affinis)
Source: Chromosome Res. 2026 Feb 20;34(1):2. doi: 10.1007/s10577-026-09793-7 (PMC12923391; doi:10.1007/s10577-026-09793-7)

Table S1. Primer sequences, annealing temperatures (ºC), and DNA template concentration (in ng/μL) used in PCR of satDNA sequences.

Satellite	Primer Fwd (5'–3')	Primer Rev (5'–3')	(ºC)	[ng/μl]
***Aaf*Sat01-200**	GCTTAGCAGTAAGGCAAAGC	AATTACCGACAGAACCATCCA	57.6	40
***Aaf*Sat02-2918**	GGCAATCGTTTTTCTCAGCT	TGCCATCAGTCGTCAACTAA	60.2	40
***Aaf*Sat03-235**	TCTGGCTGTTCTAAAGCGTT	AACGATGCAGTACAGAAGCT	57.6	2
***Aaf*Sat04-176**	TCCTAAAGCCATTCAGTCAGC	CCATACATGTTCTGGCACAAC	57.6	4
***Aaf*Sat05-1323**	GCCCGCTAAATGCTGTAAAT	GCTCTTTTCCACAGCGATTT	55.1	40
***Aaf*Sat06-343**	CTGTTCCACACCTTTGCTTT	GCCCATGAACACTAGTACGA	55.1	0,4
***Aaf*Sat07-1242**	AGCAACTCTCTGGCATTCAT	GCTCTGATGGCTAAGTTCGA	52.5	80
***Aaf*Sat08-434**	GCTCTGATGGCTAAGTTCGA	ACGGTTCACAGTTAGCATTTG	55.1	40
***Aaf*Sat10-143**	TCCACAGCACATCGCAATTA	AGCACAGTTCACGCTTTAGT	56.4	0,2
***Aaf*Sat11-227**	AAACGACATTCCAGAAGTGC	AATTCAACGCACTGAGCAAT	57.6	4
***Aaf*Sat13-158**	TGGAGACAGTGAAGTGCTTT	AGGCTTAGTGTTGAGTTACGT	56.4	0,8
***Aaf*Sat14-342**	TACACACTAATCTCGGCCCT	GGGTCCCGCCATTTAAACAT	56.4	40
***Aaf*Sat15-814**	TTCTGTGACGCTATGTTGGA	TCTTGCTCAGAGGACATCAG	55.1	40
***Aaf*Sat16-1702**	GTCCACTCAATGAAAGCACC	TATTCTTGTGGCTTGCTTGC	60.2	40
***Aaf*Sat17-217**	GTGAACTGGGAGAGTCTGG	TGTCACGCTCACCATTAAGT	58.9	40
***Aaf*Sat18-179**	CAACTCTGACCACCGACTAA	GCTTTGGACCTCCCTAAGTT	56.4	20
***Aaf*Sat19-214**	AAGTGACTCTGCCCAAAACA	GCCTTCCTCCATTTTCCTTC	57.6	20

Table S2. Sampling data for *A. affinis* from the Lower Paraná River system, including hydrographic basin, geographical coordinates, number of specimens (N), and deposit record (Voucher) in the Ichthyology Museum.

Lower Paraná River karyomorphs	River	Geographical Coordinates	N	Voucher
Karyomorph B	Uruguay	27º04'40'' S<br>53º00'14'' W	(9 ♀; 8 ♂)	NUP16270
Karyomorph C	Cuiabá	15º35'45'' S<br>56º05'49'' W	(6 ♀; 5 ♂)	NUP16265
Karyomorph D	Paraguay	16º04'16'' S<br>57º40'44'' W	(9 ♀; 7 ♂)	NUP16264

NUP = Núcleo de Pesquisas em Limnologia, Ictiologia e Aquicultura - Universidade Estadual de Maringá

Figure S1. Metaphases plates of *A. affinis* from the Uruguay River – karyomorph B (a, d, g), Cuiabá River – karyomorph C (b, e, h), and Paraguay River – karyomorph D (c, f, i) subjected to FISH with the probes *Aaf*Sat04-176 (a, b, c); *Aaf*Sat05-1323 (d, e, f); *Aaf*Sat06-342 (g, h, i). Bar = 10 µm.

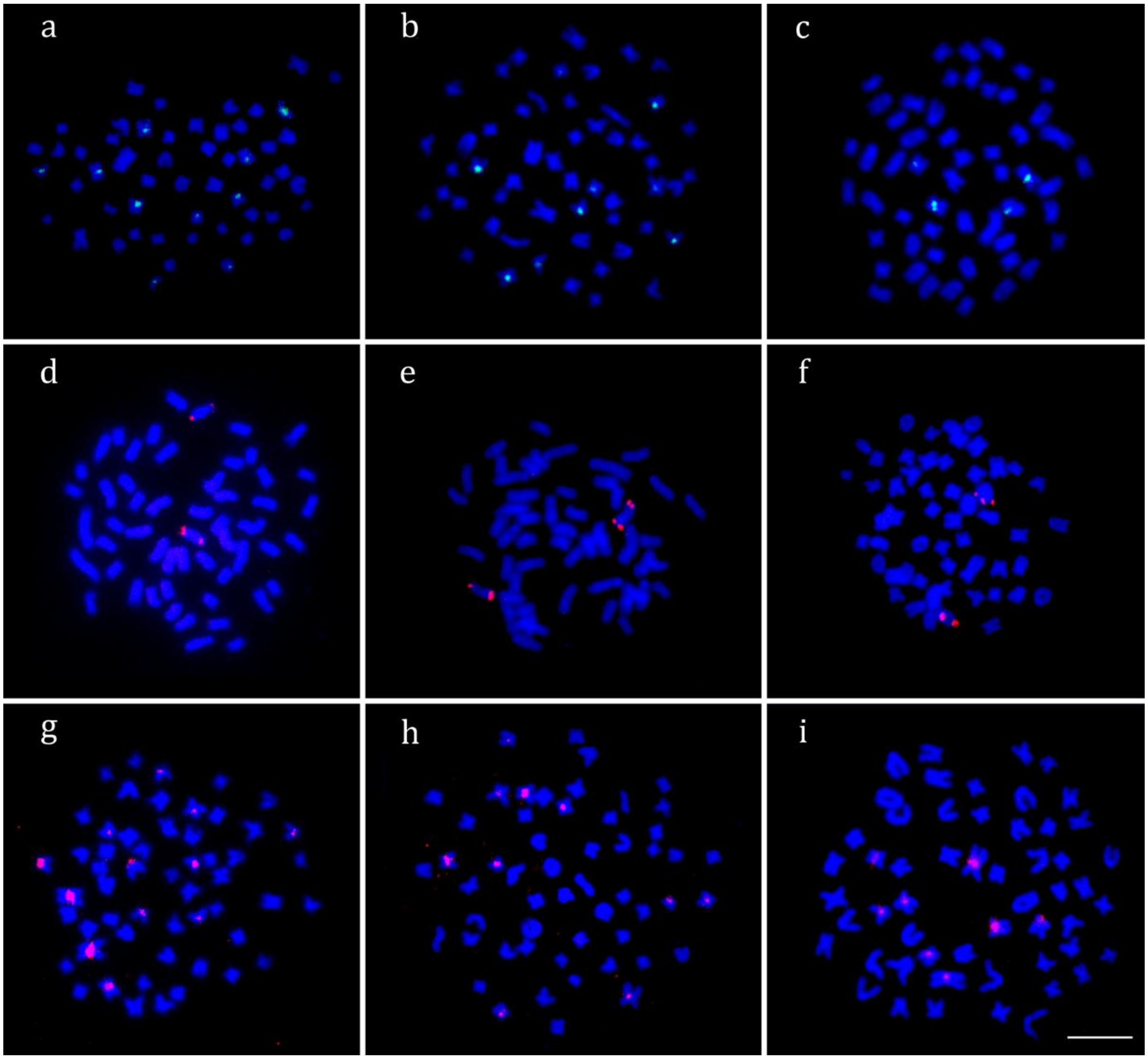

Figure S2. Metaphases plates of *A. affinis* from the Uruguay River – karyomorph B (a, d, g), Cuiabá River – karyomorph C (b, e, h), and Paraguay River – karyomorph D (c, f, i) subjected to FISH with the probes *Aaf*Sat08-434 (a, b, c); *Aaf*Sat10-1323 (d, e, f); *Aaf*Sat12-52 (g, h, i). Bar = 10 μm.

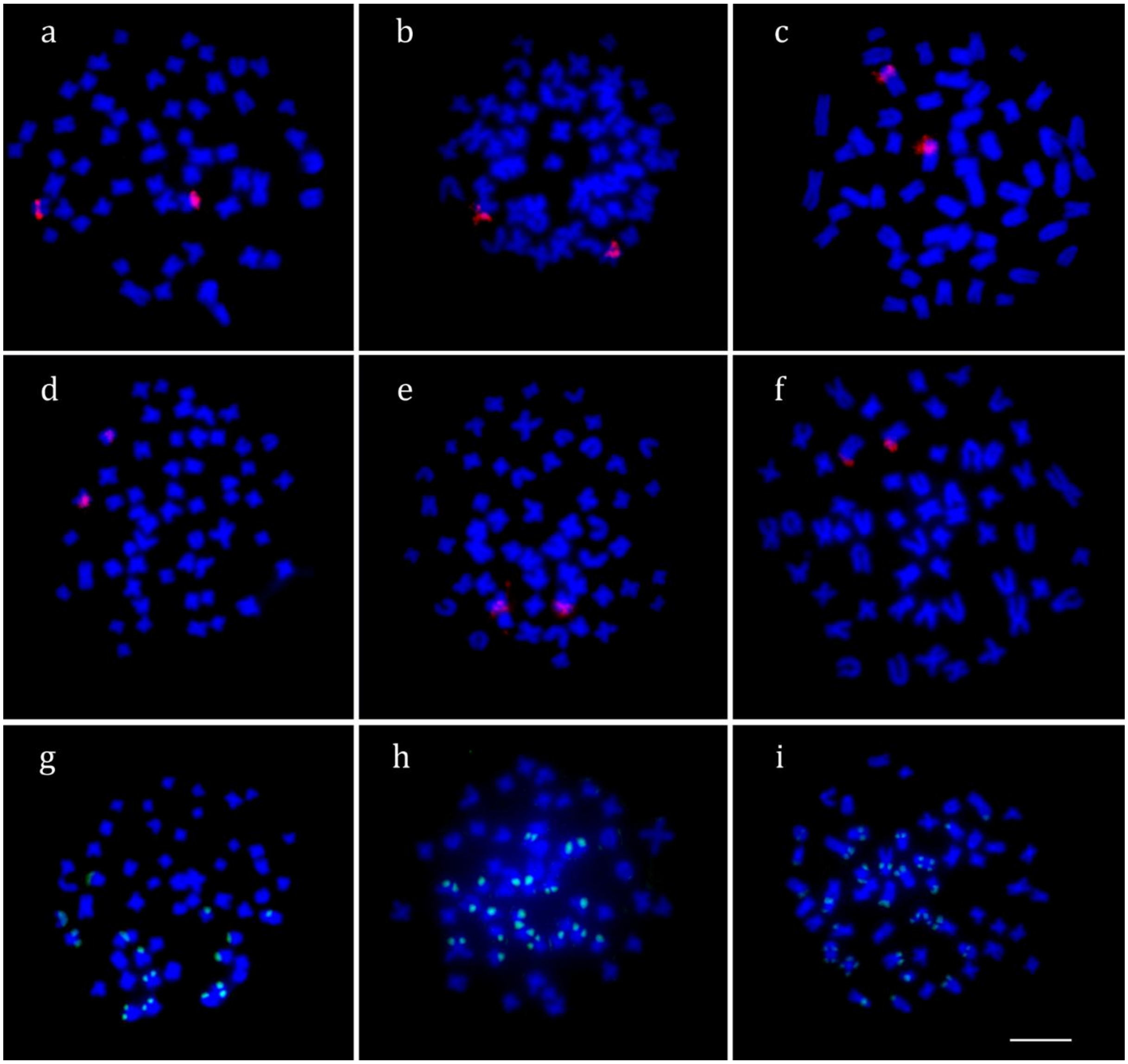

Figure S3. Metaphases plates of *A. affinis* from the Uruguay River – karyomorph B (a, d, g), Cuiabá River – karyomorph C (b, e, h), and Paraguay River – karyomorph D (c, f, i) subjected to FISH with the probes *Aaf*Sat13-158 (a, b, c); *Aaf*Sat15-814 (d, e, f); *Aaf*Sat16-1702 (g, h, i). Bar = 10 μm.

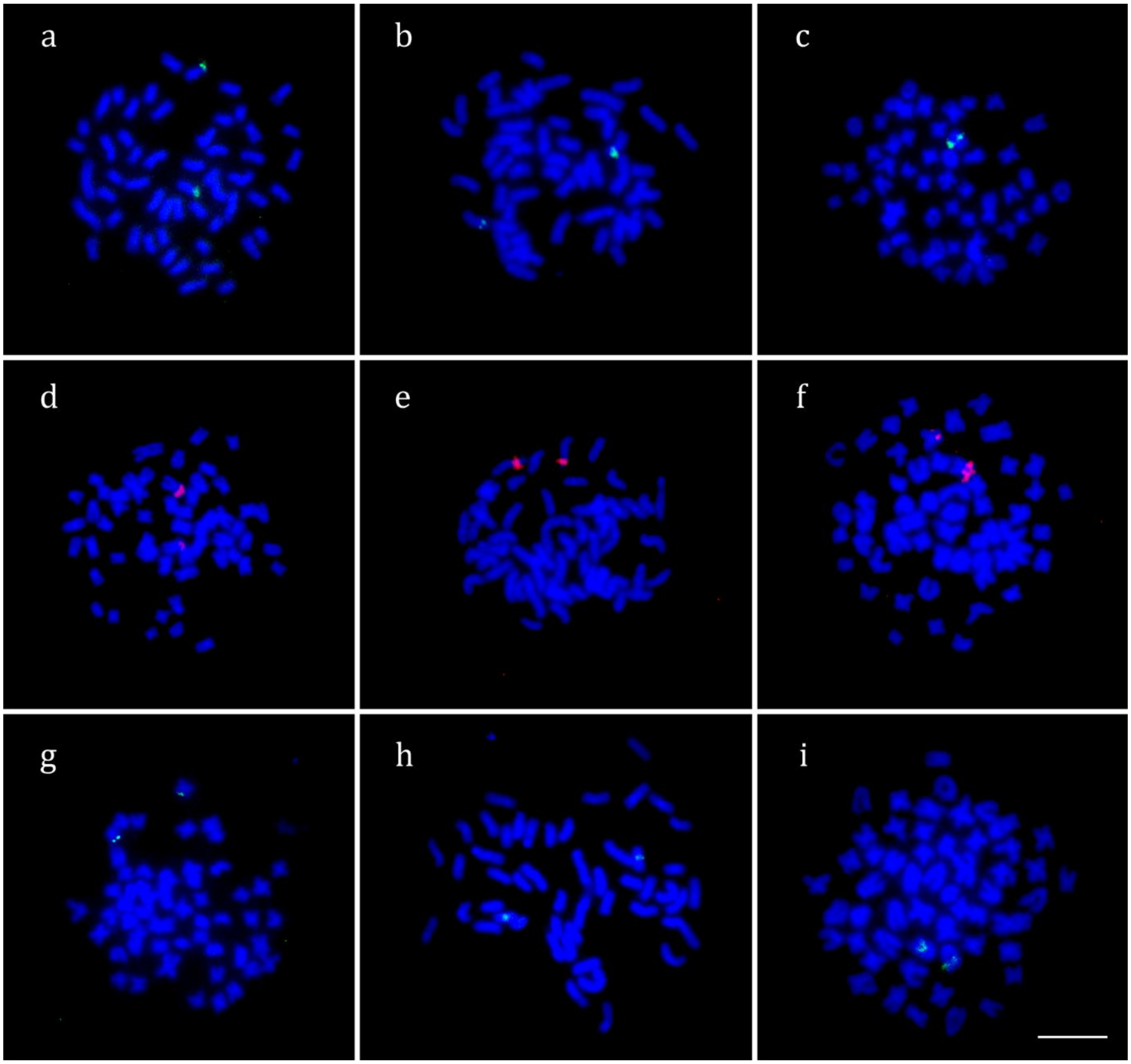

Figure S4. Metaphases plates of *A. affinis* from the Uruguay River – karyomorph B (a, d, g), Cuiabá River – karyomorph C (b, e, h), and Paraguay River – karyomorph D (c, f, i) subjected to FISH with the probes *Aaf*Sat17-217 (a, b, c); *Aaf*Sat18-179 (d, e, f); *Aaf*Sat19-214 (g, h, i). Bar = 10 µm.

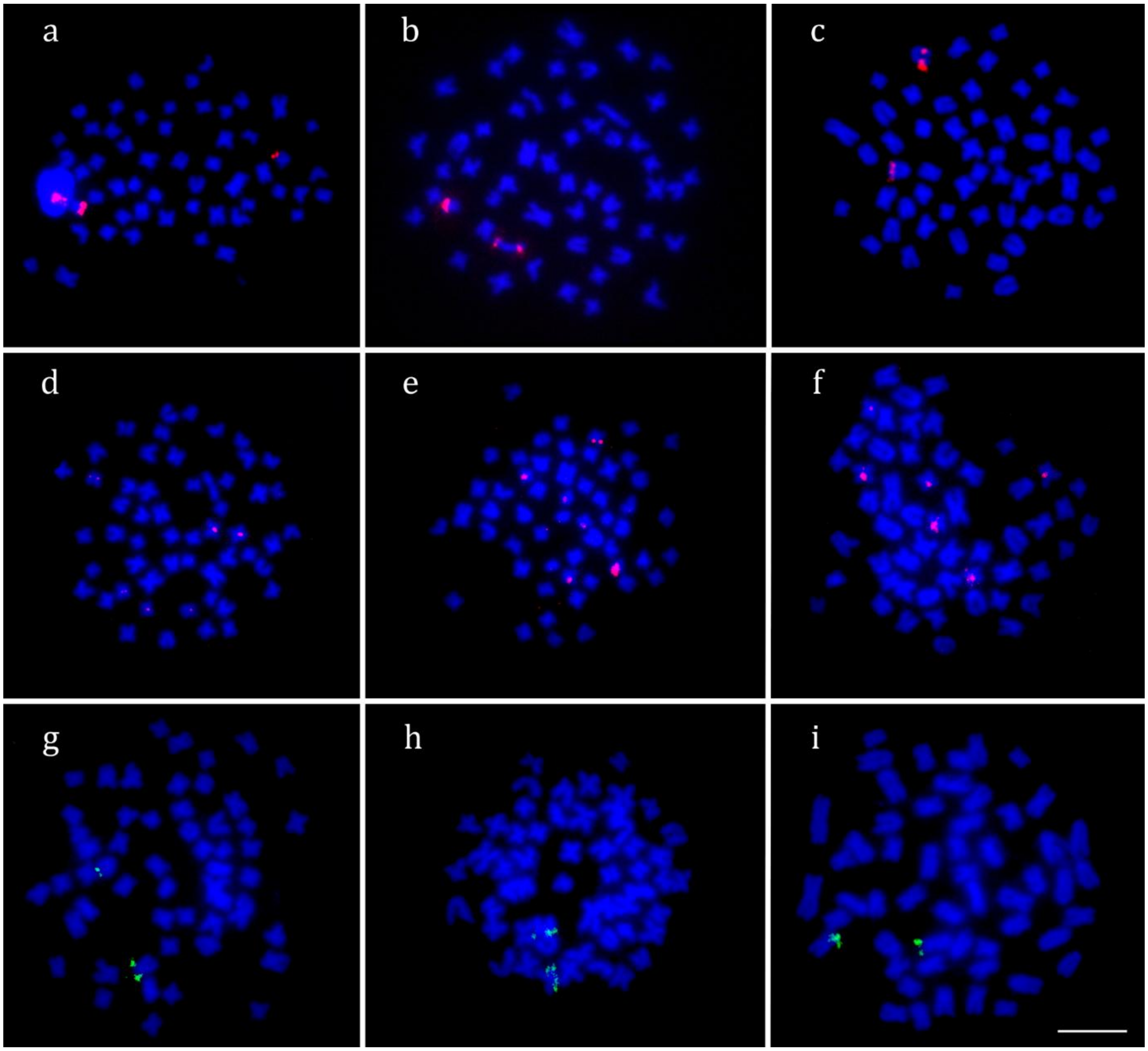

Supplement: Supplementary file 1 — Supplementary file1 (PDF 580 KB) [file 10577_2026_9793_MOESM1_ESM.pdf]
